# Practices of strength and conditioning coaches in Brazilian elite soccer

**DOI:** 10.5114/biolsport.2022.108703

**Published:** 2021-10-06

**Authors:** Irineu Loturco, Tomás T. Freitas, Pedro E. Alcaraz, Ronaldo Kobal, Renan F. Hartmann Nunes, Anthony Weldon, Lucas A. Pereira

**Affiliations:** 1 NAR – Nucleus of High Performance in Sport, São Paulo, Brazil; 2 Department of Human Movement Science, Federal University of São Paulo, São Paulo, Brazil; 3 University of South Wales, Pontypridd, Wales, United Kingdom; 4 UCAM Research Center for High Performance Sport – Catholic University of Murcia, Murcia, Spain; 5 Faculty of Sport Sciences – Catholic University of Murcia, Murcia, Spain; 6 Sport Club Corinthians Paulista, São Paulo, Brazil; 7 AFE – Associação Ferroviária de Esportes, Ferroviária, Brazil; 8 Human Performance Laboratory – The Technological and Higher Education Institute of Hong Kong (THEi), Hong Kong

**Keywords:** Athletic performance, Football, Team-sports, Coaching practices, Resistance training

## Abstract

Brazil is the leading global exporter of soccer players, with approximately 2,000 international transfers to different clubs per year. Although Brazilian players compete in the most prestigious soccer leagues worldwide, the habitual training methods, strategies, and routines of Brazilian soccer strength and conditioning coaches (SCCs) are undocumented. This study used a standard online survey to collect and characterize the strength and conditioning practices of Brazilian soccer SCCs. Forty-nine SCCs (age: 40.4 ± 7.5 years; professional experience: 15.3 ± 7.5 years) working in Brazilian professional soccer teams participated in this study. The survey consisted of eight sections: 1) background information; 2) muscular strength-power development; 3) speed training; 4) plyometrics; 5) flexibility training; 6) physical testing; 7) technology use; and 8) programing. Results indicated that training and testing practices of Brazilian SCCs are strongly affected by the congested fixture schedules, extensive traveling distances, and socio-economic disparities between different regions of the country. We describe all these different strategies and methods in detail, providing a comprehensive view and a critical examination of Brazilian soccer strength and conditioning practices. Brazilian SCCs and professional soccer organizations can use the findings from this study to develop training strategies and customize education programs. Practitioners from other countries can use this information to design training programs closely tailored to the background of Brazilian athletes, which may support their adaptation to different competitive scenarios and game demands, such as those found in the most important soccer leagues worldwide.

## INTRODUCTION

Brazil is a continental-sized country with huge socio-economic disparities between cities and regions, but with a strong and common feeling: the passion for soccer [1, 2]. Among the twenty-seven states and at the National level, there are normally 10 official tournaments competed for annually, including senior players across four divisions [3]. This large number of competitions includes a high number of professional soccer clubs (i.e., 742 in 2019) [4], resulting in a vast number of soccer players and coaches registered with the Brazilian Football Confederation (CBF) (i.e., 22,177 professional contracts and 477 soccer coaches registered with the CBF in 2019) [4]. Furthermore, to date, Brazil is the most succesful nation in the FIFA World Cup history, with a total of five world titles. Collectively, these unique and singular features contribute to making Brazil one of the biggest producers and exporters of soccer players in the world [5, 6].

In 2019, for instance, Brazil was the leading global exporter of elite soccer players, with about 2,000 international transfers to different clubs worldwide, amounting to more than $370 million in negotiations [7]. Comparatively, Argentina- the second largest global exporter of soccer players – oversaw 946 international transfers in 2019, which equates to less than half of the Brazilian soccer market [7]. This expressive number of transfers results in a great number of Brazilian athletes playing in the major soccer leagues around the world, which are consistently characterized by their high and ever-increasing physical and physiological demands [8–10]. For example, Barnes et al. [8] reported across seven successive seasons of the English Premier League that the distance covered through sprinting and high-intensity running increased by ~35%, which is very similar to that reported in other studies [9, 11]. Undoubtedly, these current trends regarding the physical, technical, and tactical demands in elite soccer pose significant challenges to soccer coaches and technical staff, especially concerning the implementation of effective and time-efficient strength and conditioning practices [12–14].

Despite these facts, there is no information in the literature about the training methods, schemes, or types of exercise most frequently used by Brazilian soccer strength and conditioning coaches (SCCs). These data are important to better understand and refine Brazilian regional practices in strength and conditioning and provide the international soccer community with an integrated and holistic view of how Brazilian players are trained and tested in Brazilian clubs. This would allow the creation of optimal training strategies for adapting and developing Brazilian soccer players, according to the specific requirements of the most prestigious international soccer leagues. Therefore, this study aimed to describe the training and testing procedures commonly employed by SCCs working in the Brazilian soccer scenario.

## MATERIALS AND METHODS

### Participants

Forty-nine Brazilian soccer SCCs (age: 40.4 ± 7.5 years; age range: 25–58 years; professional experience: 15.3 ± 7.5 years; range: 3–31 years), who worked in division 1 (53.1%), division 2 (28.6%), division 3 (6.1%), and division 4 (12.2%) of the Brazilian National Championship completed the survey. Regarding the academic background of SCCs, 12% held a PhD degree, 27% a master’s degree, 49% had already completed a post-graduate course, and all of them were graduated in Physical Education or Sport Science. The study was conducted under the ethical standards of the Helsinki Declaration and was approved by the local Ethics Committee.

### Study Design

This cross-sectional descriptive study was designed to characterize the common training and testing practices of Brazilian soccer SCCs. Given that these SCCs are commonly encouraged to implement contemporary research and science-informed practices, it is important to establish if this is the case. For this purpose, a survey used in a previous study to assess the practices of SCCs [14] was adapted and designed using Typeform^TM^. The survey consisted of eight sections (1-background information; 2-muscular strength and power development; 3-speed development; 4-plyometrics; 5-flexibility; 6-physical testing; 7-technology use; and 8-programing) comprising 27 fixed responses and 15 open-ended questions. Some questions allowed more than one response, meaning that some questions have more responses than others. During the survey preparation, four experienced SCCs completed the survey and minor adjustments were made to the wording and structure of some questions, to ensure they were clear and appropriate for the surveyed population. A complete explanation of the general information necessary to complete the survey, the study purpose, and the confidentiality of information and identity were provided on the first page. Thereafter, participants provided consent and anonymously completed the online survey.

### Data Acquisition and Analyses

The survey responses were downloaded from Typeform^TM^ into a customized spreadsheet. Fixed response questions were assessed using a frequency analysis and open-ended questions using a thematic analysis approach [15], via the following process: 1) familiarization with the data, 2) generating initial codes, 3) searching for themes, 4) reviewing themes, 5) defining and naming themes, and 6) producing the report. This thematic-analysis method has been used in prior studies surveying SCCs [14, 16]. Subsequently, key themes representing the main ideas emerging from the raw data were generated for open-ended questions. Some responses provided sufficient information that multiple topics could be identified and considered for further analysis. All topics were reviewed and agreed by all authors.

## RESULTS

### Muscular Strength and Power Development

**FIG 1:**
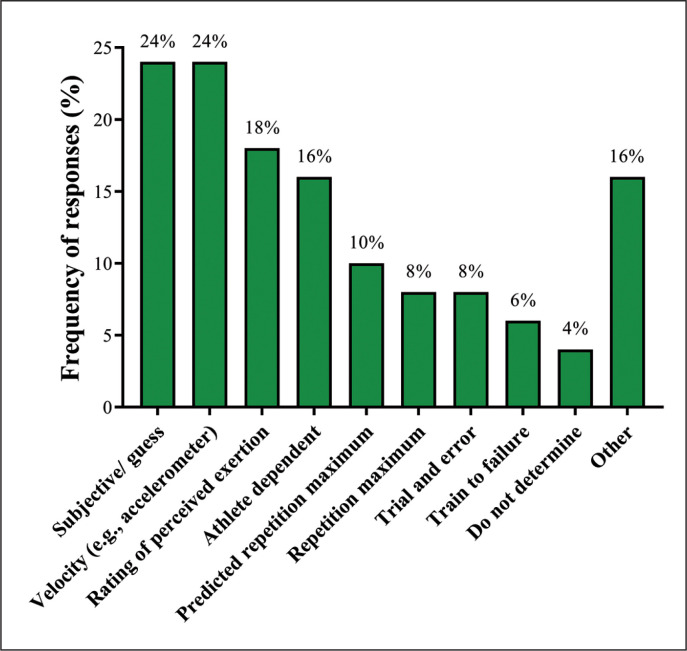
Load determination procedures used during resistance training sessions by Brazilian strength and conditioning coaches.

Table 1 shows the frequency of responses regarding the organization of strength training programs during preparatory and competitive periods. Figure 1 depicts responses to how SCCs determined set loads during strength training sessions. Table 2 demonstrates absolute and relative results regarding the use or not of periodization strategies in their strength training program. Table 3 shows the average recovery time prescribed between competitive matches, soccer-specific training, and strength/power training sessions. Additionally, SCCs were asked whether they use Olympic weightlifting and associated derivates in their programs. Responses demonstrated that most SCCs did not use Olympic weightlifting exercises (71%), whereas 20% used the clean and jerk, 18% snatch, 8% clean, 4% push press, and 2% snatch high pull. Moreover, SCCs were asked what methods of resistance they implement within their programs, with the most commonly reported methods being eccentric (67%), concentric (65%), variable (59%), machine (37%), isometric (27%), and isoinertial (24%). Table 4 demonstrates the ranking of the five most important exercises that SCCs used in their strength training programs.

**TABLE 1:** Absolute (and relative, %) frequency of responses regarding the organization of the strength training program during preparatory (PP) and competitive (CP) periods (n = 49).

Number of sessions/week		**1**	**2**	**3**	**4**	**5**	**+5**	**Other**
	PP	0(0)	15(31)	24(49)	7(14)	3(6)	0(0)	0(0)
	CP	8(16)	34(70)	3(6)	3(6)	0(0)	0(0)	1(2)

Session length (minutes)		**0–15**	**16–30**	**31–45**	**46–60**	**61–75**	**+75**	**Other**
	PP	1(2)	13(27)	24(49)	9(18)	1(2)	1(2)	0(0)
	CP	3(6)	27(55)	13(27)	4(8)	2(4)	0(0)	0(0)

Number of sets for each exercise		**1–2**	**3–4**	**5–6**	**7–8**	**9–10**	**+10**	**Other**
	PP	6(12)	31(63)	4(8)	4(8)	2(4)	0(0)	2(4)
	CP	20(41)	19(39)	7(14)	1(2)	1(2)	0(0)	1(2)

Number of repetitions for each exercise		**1–3**	**4–6**	**7–9**	**10–12**	**13–15**	**+15**	**Other**
	PP	1(2)	17(35)	20(41)	8(16)	0(0)	0(0)	3(6)
	CP	0(0)	25(51)	18(37)	4(8)	0(0)	0(0)	2(4)

**Table 2:** Responses regarding models of the strength training plan over the season (n = 49).

	Absolute (n)	Relative (%)
Through the use of periodization models that follow preplanned and/or fixed routines, selecting some matches as “most important matches” where players must achieve peak performance	3	6
Through the use of programs constantly readjusted according to the individual or collective physical/physiological responses, not necessarily following fixed routines, trying to maintain high performance levels during all matches of the championship	46	94

**Table 3:** Absolute (and relative, %) frequency of responses regarding the average recovery time between distinct sessions (n = 49).

	Same day	24 h	36 h	48 h	> 48 h
Recovery time between strength/power training and soccer-specific training	31(63)	8(16)	4(8)	6(12)	0(0)
Recovery time between strength/power training and match	1(2)	5(10)	7(14)	14(29)	22(45)

**Table 4:** Ranking of the five most important exercises used in the strength training programs (n = 49).

Order of importance	Exercises	n (%)
1	Squat and variations Hip Thrust Leg Curl Nordic Stiff-leg deadlift Did not specify	35(71) 3(6) 1(2) 1(2) 1(2) 8(16)
2	Squat and variations Hip Thrust Nordic Lunge Stiff-leg deadlift Did not specify	17(35) 10(20) 7(14) 4(8) 4(8) 7(14)
3	Squat and variations Stiff-leg deadlift Hip Thrust Lunges Resisted sprint Jumps Nordic Copenhagen Leg Curl Leg Press Did not specify	11(22) 7(14) 6(12) 5(10) 4(8) 3(6) 3(6) 1(2) 1(2) 1(2) 7(14)
4	Squat and variations Lunges Stiff-leg deadlift Leg Curl Copenhagen Jumps Resisted Sprint Hip Thrust Nordic Snatch Did not specify	9(18) 8(16) 6(12) 4(8) 3(6) 2(4) 2(4) 1(2) 1(2) 1(2) 12(24)
5	Squat and variations Lunges Hip Thrust Jumps Nordic Stiff-leg deadlift Calf Rise Leg Curl Leg Press Did not specify	9(18) 8(16) 3(6) 3(6) 3(6) 3(6) 2(4) 2(4) 2(4) 14(29)

### Speed Development

**FIG. 2:**
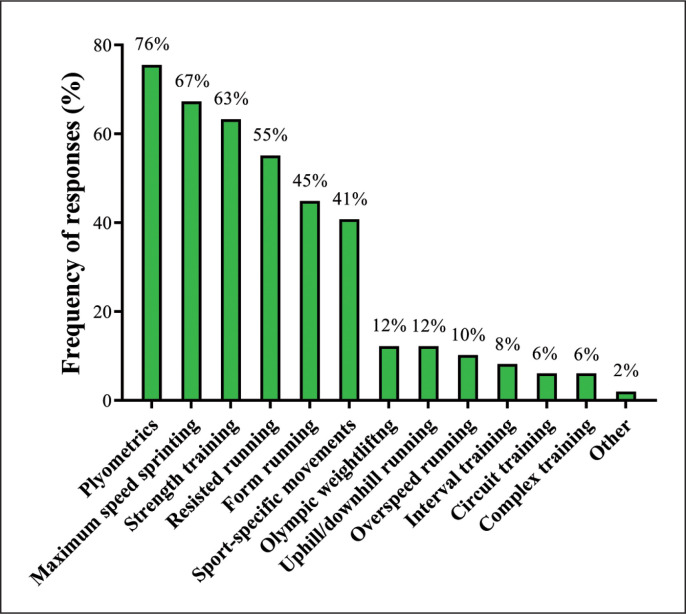
Methods for speed development used by Brazilian strength and conditioning coaches.

Figure 2 depicts the responses regarding the methods most commonly used by SCCs for speed development. The methods most frequently reported were plyometrics (76%), maximum speed sprinting (67%), and strength training (63%).

### Plyometrics

The SCCs were asked the main reasons why they implemented plyometric exercises in their programs, with speed development (86%) being the most reported, followed by improving jump ability (55%), injury prevention (51%), lower-body power (47%), total-body power (4%), and upper-body power (2%). Two SCCs (4%) reported other reasons, including*“improve muscular coordination”, “improve muscular power”, and “stretch and shortening-cycle optimization”*.

**FIG. 3:**
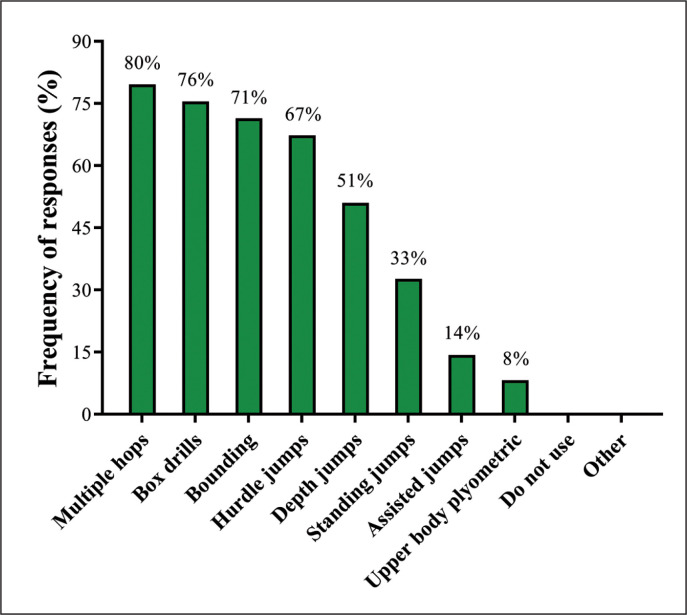
Plyometric exercises used by Brazilian strength and conditioning coaches.

Regarding the period of the season that SCCs usually employ plyometric training, pre-season (55%) and in-season (53%) stages were the most frequently reported, followed by all year round (41%), off-season (2%), and other (2%). Regarding the integration of plyometrics into their training schedule, 49% of the SCCs reported that it is used as part of complex training, 31% after weight training, 27% before weight training, 22% on separate days, and 4% selected the option other. Figure 3 depicts the frequency of responses concerning plyometric exercises commonly used by SCCs in their programs.

### Flexibility Development

Soccer SCCs were asked to report when players were encouraged or required to perform flexibility exercises in their program. Before training (49%) was the most frequent option followed by after training (16%), during training (12%), independently/on their own (10%), other (4%), and do not use (27%). The most common forms of flexibility training used by SCCs were dynamic (55%), ballistic and active (39%), proprioceptive neuromuscular facilitation (27%), passive (20%), isometric (4%), static (2%), and did not use (18%). The average duration of a typical flexibility session was 6–10 minutes (33%), 0–5 minutes (20%), 11–15 minutes (16%), and 16–20 minutes (4%), whereas 20% did not perform flexibility sessions.

### Physical Testing

**FIG. 4:**
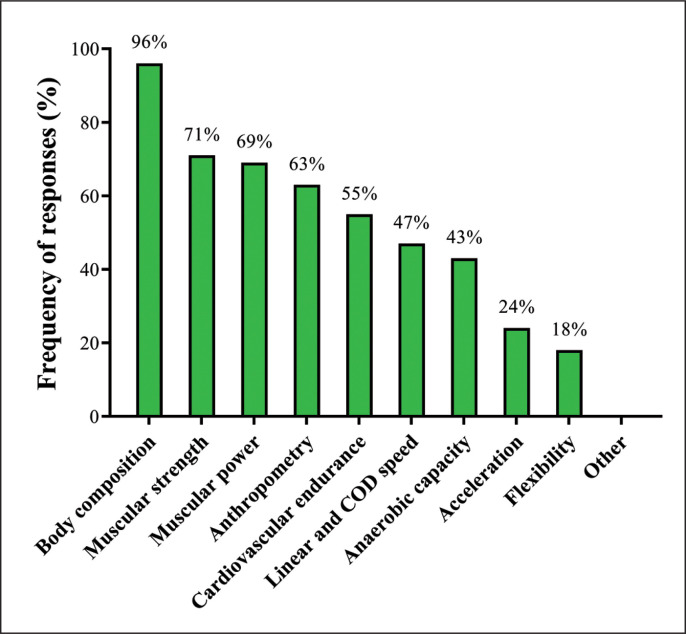
Physical tests employed by Brazilian strength and conditioning coaches. (*COD: change of direction*).

The most common time reported by SCCs for physically testing players was all year round (53%), followed by during the pre-season (47%), in-season (22%), and do not test (4%). Figure 4 shows the frequency of responses regarding the physical tests commonly used by SCCs with their players. Furthermore, SCCs were asked how they monitored player’s well-being, with online questionnaires or mobile applications (45%) being the most reported, and verbal questionnaires (43%) or written questionnaires (31%) also commonly used.

### Technology Use

**FIG. 5:**
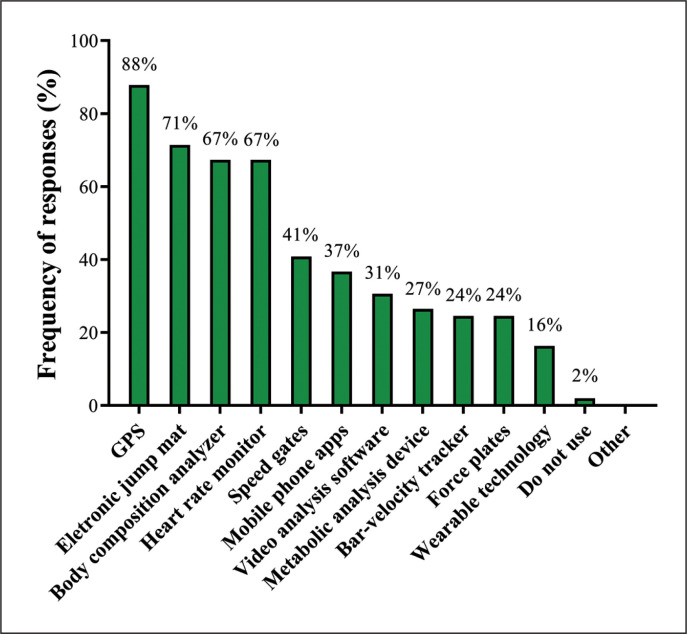
Technology-based equipment utilized by Brazilian strength and conditioning coaches.*(GPS: global positioning system)*.

Figure 5 depicts responses regarding the technology-based equipment that SCCs used in their training programs. Almost 90% of them used global positioning systems (GPS) and 71% used electronic jump mats. Other frequent responses were heart rate monitor and body composition analyzer (both 67%).

### Programming

The SCCs were asked to report the biggest issues faced as a SCC. Responses included congested fixtures (41%), poor professional relationship with coach/players (20%), inadequate equipment/facilities (12%), and individualizing training loads (6%). Other responses (21%) comprised a “*high number of players move to other teams”*, “*high pressure for immediate results”*, and “*unexpected changes in the match schedule”*. Another question was to report if SCCs felt there was anything unique about their training program, with most SCCs reporting “no” (63%), while 37% answered “yes”. Some affirmative responses were: *“an interpersonal relationship with team-members”, “interdisciplinary approach”, “training load control and integration with technical and tactical training”, “the strength training methodology”.* Also, SCCs were asked if they employed strategies to individualize training loads according to the playing positions and characteristics of each player, with 93% answering “yes” and 6% “no”. When asked if they would change something in their training program, given unlimited time and resources, 84% answered “yes”, while 16% answered “no”. For the SCCs who selected “yes”, some responses involved better training individualization, use of more equipment (technology), and an increase in the frequency of strength training sessions during the week. Finally, the last question was about their opinion on future trends in strength and conditioning, with training individualization (31%) being the most reported, followed by technology (24%), higher frequency of strength training (10%), and use of artificial intelligence and machine learning techniques (8%). Other responses included real-time training monitoring, better integration between science and practice, and increased training specificity.

## DISCUSSION

This is the first study to describe the training and testing practices of SCCs working in Brazilian professional soccer. The data presented here allow deeper and more detailed understanding of the habitual training methods, strategies, and routines adopted by these professionals. These findings may be useful and relevant not only for regional and national purposes, but also for the international soccer community.

### Resistance Training Prescription

#### Preparatory Period

During the preparatory period (i.e., pre-season phase), most SCCs prescribed 2–3 resistance training sessions per week (31% and 49% of the SCC, respectively). Less than 20% of SCCs implemented 4–5 sessions per week, with none applying 1 session per week. A typical training session lasts, on average, less than 45-min for 78% of SCCs. A considerable part of these professionals (22%) prescribe training sessions longer than 45-min, with a small minority utilizing sessions shorter than 15-min (2%) or longer than 60-min (4%). Most (63%) use 3–4 sets per exercise; approximately 20% and 12% of them use 5–10 sets or 1–2 sets per exercise, respectively. The number of repetitions per set ranges from 4–6 to 7–9 in a very balanced manner (35% versus 41%, respectively), although ~16% of the SCCs implement a range of 10–12 repetitions. None of the SCCs prescribe sets with more than 12 repetitions (13–15 repetitions) and only 2% of them prescribe sets with less than 4 repetitions (1–3 repetitions). In general, these training settings (i.e., 2–3 sessions lasting ≤ 45-min per week, 3–4 sets of ≤ 10 repetitions per exercise) are in line with the body of literature concerning resistance training prescription during short and congested soccer pre-seasons (i.e., 4–6 weeks) [12, 13, 17]. Studies with elite soccer players commonly report a high number and frequency of soccer-specific activities per week (e.g., small-sided games and friendly matches), which also imposes a large aerobic demand on these athletes, possibly compromising effective speed and power development [12, 18]. Therefore, the adoption of training schemes with short (≤ 45 min) but frequent (2–3 sessions) resistance training sessions per week seems to be a viable alternative to avoid (or at least minimize) the inhibitory effects of concurrent training on neuromuscular adaptations [12, 13, 18]. Nevertheless, investigations with Brazilian soccer players have consistently shown that this volume of resistance training may not be sufficient to improve the physical performance of these athletes, but instead may only counteract the speed-power decrements which commonly occur during high-volume soccer pre-seasons [12, 19]. Thus, it is likely that this training regimen should be revisited and adjusted, especially if the main objective is to produce faster and more powerful players, which appears to be a tendency in modern soccer [8, 19, 20]. In this regard, and considering the inherent limitations imposed by short soccer pre-seasons, a small (but appropriate) increase in the frequency of strength-power training sessions can be highly recommended [21].

#### Competitive Period

In general, as expected, it is possible to observe an important reduction in the total volume of resistance training during the in-season phase. About 85% of SCCs prescribe 1(16%) or 2 (69%) resistance training sessions per week and only a minority implement 3 or 4 sessions per week (6% for both). Likewise, the average training time is substantially reduced (in comparison to pre-season), with 55% of SCCs adopting resistance training sessions lasting from 16- to 30-min, and 27% prescribing training sessions of 31–45-min. Less than 18% of SCCs utilize training sessions of 0–15, 46–60, and 61–75 min (6%, 8%, and 4%, respectively). Similar to the preparatory period, a higher proportion of SCCs continue to use 3–4 sets per exercise (39%), although a significant increase in the number of SCCs using 1–2 sets in their training routines was noted (41% versus 12% in the pre-season phase). Curiously, the number of repetitions per set also tends to remain stable when compared to the preparatory period, with 51% and 37% of the SCCs prescribing sets with 4–6 and 7–9 repetitions (against 35% and 41%, respectively, in the pre-season phase). Lastly, 8% of SCCs implement sets with 10–12 repetitions and none of them use sets with 1–3 repetitions during this period. Based on these observations, it is possible to deduce that this training arrangement is almost exclusively related to the need to reduce the total training load across this period, which naturally presents a high volume of technical-tactical sessions and matches [12, 22]. Therefore, the overall reduction in the resistance training content seems to be an alternative strategy adopted by SCCs to counterbalance the significant and progressive increase in soccer-specific training content, which inevitably occurs throughout this phase [23–26]. In practical terms, SCCs are perceivably attempting to maintain the strength and power training practices in their training schedule, while reducing the non-specific training load to avoid (or at least minimize) the detrimental effects of excessive fatigue on player performance [27, 28]. Again, this could be a problematic issue, as it has been revealed that neuromuscular control tends to decrease over a competitive season [29, 30], which, among other things, may provoke a plateau or even a decrease in certain speed-power qualities (e.g., vertical jumps and short-sprints) from mid to later stages of the competitive period [29, 31]. These effects may be even more pronounced during periods of congested match-play due to the accumulated fatigue, inadequate recovery time, and compromised opportunities to carry out resistance training sessions [30, 32]. Besides the possible decrements in performance, these factors have been commonly associated with greater levels of physiological stress and muscle damage and, potentially, with increased injury risk [23, 25, 33].

Although we recognize the challenges of this complex training period, these observations suggest that the continuous development of optimal levels of strength, power, and neuromuscular control may be essential to minimize the negative effects of high match exposure (i.e., cumulative fatigue associated with concurrent training) [34] throughout the in-season phase. In this sense, programs with higher volumes of resistance training would probably help practitioners reduce injury rates and performance decrements towards the end of the soccer season [35, 36]. Nevertheless, limitations in training time will always be a great barrier in modern soccer; therefore, Brazilian SCCs are advised to use all available training means and resources, such as brief warm-up activities and hybrid training sessions (e.g, circuit-training workouts including physical and technical elements and alternating heavy and light resistances in both traditional and ballistic exercises) in an attempt to significantly improve strength- and power-related capacities. Irrespective of time duration, when performed under appropriate conditions, these short and multifaceted training sessions may work as an efficient way to promote both acute and chronic gains in neuromuscular performance [37–39].

### Loading Determination and Resistance Training Programming

A similar percentage of Brazilian SCCs determine the training loads based on movement velocity (i.e., velocity-based training) and subjective guess (24% for both). The second and third most common methods were rating of perceived exertion (18%) and athlete dependent (16%). Curiously, only 10% of SCCs in this study used predicted repetition maximum (10%) or repetition maximum (8%) tests to determine set loads, which is considerably lower than the data reported by Weldon et al. [14] from SCCs (non-Brazilian) working in professional soccer in various countries. These differences may be related to different cultural perspectives and a regional background in resistance training within the Brazilian soccer context, especially the execution of maximum dynamic strength tests. Studies with Brazilian soccer players frequently highlight the inherent risks and time-consuming nature of 1RM tests (characteristics that have also been pointed out by authors in other sport disciplines) [40–42]. The remaining responses included trial and error (8%), training to failure (6%), do not determine (4%), and other (16%; e.g.,% of body-mass, 10-RM test).

Most Brazilian SCCs (63%) program strength-power sessions on the same day as soccer-specific training sessions, which is logical given the congested schedules of professional soccer clubs [23, 33]. Minor differences were observed among the other options, with 16%, 8%, and 12% of SCCs programming their training sessions with intervals of 24, 36, and 48h, respectively, until the next soccer-specific training session. When considering competitive matches, a trend towards longer intervals was observed, with 29% and 45% of SCCs using intervals greater or equal to 48 h, respectively. This is expected when considering the greater levels of fatigue experienced during a soccer match [27, 43]. About 14% of SCCs prefer to adopt intervals of 36 h, with only a minority utilizing intervals ≤ 24 h (12%). In addition to the physiological aspects associated with match-related fatigue, there is another point to be noted within the Brazilian context: Brazil is a continental-sized country, with some regional flights lasting up to 6–7 h. As such, SCCs have to deal with a variety of complex issues interrelated with extensive and recurrent journeys such as jetlag, sleep deprivation, traffic congestion, and airport transfers [44, 45]. Collectively, all these factors seriously compromise time management and training schedules. Nonetheless, concerns about time constraints are not exclusive to the Brazilian soccer scenario and have been consistently described in recent investigations conducted in different soccer leagues and tournaments [25, 46]. Therefore, gradual reductions in the frequency of strength-power training sessions throughout elite soccer seasons appears to be a global reality in contemporary soccer [14].

An important finding of this study is that only 6% of SCCs used a periodization scheme (i.e., a pre-programmed training plan with pre-programmed performance peaks) [47, 48]. The vast majority of Brazilian SCCs (94%) preferred to utilize flexible programming approaches, regularly adjusting training loads and strategies, according to individual or group responses to training and competitions. These data are in contrast to those obtained by Weldon et al. [14], who reported that 98% of soccer SCCs from 18 different countries implemented periodization models to structure training programs. These huge discrepancies may be related to a series of factors, including: 1) differences in the level of the sample, since we primarily considered SCCs from first and second divisions in our analysis (82% of the sample), who have to deal with congested fixture schedules, in both regional and national championships. This excessive number of matches precludes the implementation of fixed or even pre-planned training schemes, as different players will respond differently to varying combinations of training and match demands [25, 49]; 2) the double round-robin system adopted in the national tournaments [50, 51], in which every game has the same importance to win the title, making it impossible and inappropriate to establish a peak performance period. Also, these marked divergences could have been exacerbated by allowing SCCs to further elaborate their periodization preferences (i.e.,using programs frequently readjusted according to individual or collective physical and physiological responses, not necessarily following fixed routines, trying to maintain high levels of performance throughout the entire tournament). It is crucial to note that the survey used by Weldon et al. [14], only allowed SCCs to select either “yes”, “no”, or “other” with regards to whether periodization strategies were used (or not). As a result, SCCs who implemented a more flexible (but structured) training scheme in their professional routines may have been compelled to select the response “yes”. However, when it comes to training periodization, some theoretical constructs should be considered as key prerequisites for training application, such as the establishment of peak performance periods for targeted competitions and the division of training programs into distinct training phases, with definite purposes and objectives [47, 48]. As previously mentioned, these traditional concepts do not apply to modern soccer, especially in the Brazilian scenario [50, 51]. Based on our findings, researchers are advised to prepare questions with multiple choice answers that provide SCCs with a clearer and more specific understanding of what is being addressed when inquiring about training periodization. This is essential to gain more information about coaching practices not only in elite soccer, but in other sports with congested schedules and several competitions across the season.

### Resistance Training Modes and Exercises

Most Brazilian SCCs primarily used eccentric (67%), concentric (65%), and variable resistance training modes (59%), followed by machine (37%), isometric (27%), and isoinertial modes (24%). Despite the similarity of trends, there is a large difference that should be highlighted when contrasting our findings with those obtained by Weldon et al. [14]. While in the latter study 100% of international SCCs used concentric and eccentric training, the present data show that only ~65% of the Brazilian SCC regularly adopt these training modes in their daily practices. Nonetheless, for both coaching samples, variable resistance training is a prominent training mode, with 59–65% of SCCs using this training strategy with their athletes. Besides the effectiveness of this training mode (which was also confirmed in a recent study with Brazilian soccer players) [19], other factors could have influenced our results, including: 1) the high costs associated with the purchase of strength-training equipment (e.g., Olympic barbells, weight plates, weight-training machines, etc.), which may be necessary for performing some types of concentric and eccentric exercises (e.g., leg press, deadlift, squat-machine variations, flywheel devices, etc.), and 2) the high numbers of official matches and hence, journeys and displacements throughout the season (i.e., some Brazilian first division clubs can play up to ~80 matches per season). Although issues related to budget constraints (which is always a problem in developing countries like Brazil) may affect poorer clubs (i.e., 3^rd^ and 4^th^ division soccer clubs) to a greater extent, congested fixtures seem to be a challenge for the majority of SCCs and technical staff [23, 24]. Thus, playing either at home or away, Brazilian SCCs will always have to design and create alternative strategies to develop strength and power capabilities in their players. The low cost associated with the versatility and portability of elastic bands may therefore explain the popularity of variable resistance training among Brazilian SCCs [52, 53].

Squat and variations were shown to be the most popular exercises, which is in line with previous results obtained not only in soccer [14], but also across various competitive sports [16, 54, 55]. This finding may be attributed to a series of factors, such as the effectiveness and ease of application of squat-based movements and the great number of studies exploring this topic in soccer [17, 18, 41]. Completing the sequence, hip-thrust and lunge appear four times, and Nordic, leg curl, and stiff-leg deadlift appear twice among the four most common types of exercise within the five coaching ranks (Table 4). Another interesting finding is that 71% of the Brazilian SCCs declared not using Olympic weightlifting and associated derivatives in their training practices, which is also in contrast to the data reported by Weldon et al. [14], which showed that 67% of SCCs from different countries and soccer leagues prescribe these exercises. Again, these divergencies may be related to cultural practices and differences in training background [54, 55], as Brazilian soccer players do not usually perform Olympic weightlifting during their specialization years [56, 57]. Furthermore, some recent studies have indicated that less complex exercises (i.e., loaded jump squats) may be more beneficial than some specific types of weightlifting derivatives (i.e., Olympic push-press) for enhancing the speed and power performance in Brazilian soccer players [58, 59]. These issues might explain, to some extent, the data and differences in training practices between Brazilian and international SCCs reported here.

### Speed Training Strategies

As the main strategies for improving speed performance, Brazilian SCCs primarily utilize plyometrics, maximum speed sprinting, and strength training (76%, 67%, and 63% of SCCs, respectively) (Figure 2). The other most commonly used strategies are resisted running (55%), form running (e.g., skipping, backward running, high knee runs) (45%), and sport-specific movements (41%). In total, ≤ 12% of SCCs implement alternative methods for developing speed qualities such as uphill and downhill running, overspeed running, and Olympic weightlifting. These findings are very similar to those described in previous studies analyzing strength and conditioning practices in professional soccer and other sports [14, 16]. These tendencies in speed training are certainly influenced by: 1) the very high specificity of sprinting technique and mechanics, which requires SCCs to use the most specific training methods (i.e., maximum sprints and running technique) to properly enhance speed-related qualities [60–62], and 2) the positive effects of both strength and plyometric training interventions on speed performance, which were consistently observed in numerous investigations involving soccer players from different age-categories and performance levels [35, 41, 59, 63]. Lastly, the fact that a substantial number of SCCs (55%) prescribe resisted sprints may also be due to the reasons mentioned above, such as: 1) these exercises provide a specific mechanical overload when properly prescribed, allowing athletes to mimic unloaded sprints with an added resistance [64, 65], and 2) several recent studies have demonstrated the effectiveness of this training strategy on the speed performance of soccer players [17, 64, 66]. In general, the speed training strategies used by Brazilian soccer SCCs are aligned with the strategies adopted by SCCs from different countries and can be considered as evidence-based practices.

### Plyometric Training Strategies

Plyometric exercises are mostly prescribed for speed development by 86% of Brazilian SCCs. Improving jumping ability, injury prevention, and increasing lower-body power were ranked in 2^nd^, 3^rd^, and 4^th^ places of training priority in a balanced manner, being selected by 55%, 51%, and 47% of the SCCs, respectively. A comparable portion of the coaches use plyometrics during pre-season (55%) and in-season (53%) stages, and 41% of them implement this strategy all year round. Plyometrics are prescribed as part of complex training by 49%, after weight training by 31%, before weight training by 27%, and on separated days by 22% of the SCCs. Despite some similarities in plyometric training integration between Brazilian and international SCCs [14], a particular point should be highlighted and potentially criticized. A greater proportion of Brazilian SCCs (31% versus 27% for international SCCs [14]) usually prescribe plyometric exercises after weight training sessions which, in theory, may affect training quality [67, 68]. It is crucial to note that plyometric training places a high demand on the neuromuscular system, being more efficient when prescribed under well-rested (or at least not fatigued) conditions [69, 70]. This may be even more critical in modern soccer, as the time constraints imposed by congested fixture schedules require coaches to remain up-to-date and utilize the most effective training practices, especially for the development of speed- and power-related qualities [13, 18, 19, 63]. Higher proportions of Brazilian coaches prescribe multiple hops (80%), box drills (76%), bounding (71%), hurdle jumps (67%), and depth jumps (51%), while other exercises such as standing jumps, assisted jumps, and upper-body plyometrics are used by 33%, 14%, and 8% of the SCCs. In addition to their effectiveness, the high usage of plyometrics in Brazilian soccer seems also to be influenced by their low-cost (i.e., cheap and accessible materials such as plastic cones, wood boxes, and hurdles), easy applicability, and sport-specific nature [71, 72]. In summary, Brazilian SCCs frequently use different types of plyometric exercises, with different purposes and objectives, in their professional routines. Although 76% of SCCs prescribe these exercises as part of complex training and before weight training, 31% of them still prefer to utilize these exercises after weight training. These practices should be revisited so that they are in agreement with plyometric guidelines and to improve training quality [35, 69, 70].

### Flexibility Training Strategies

Approximately 50% of Brazilian soccer SCCs prescribe flexibility exercises before training, while 16% and 12% implement these exercises after and during training, respectively. Curiously, a large number of coaches (27%) declared not using any type of flexibility exercises in their training practices. To some extent, this occurrence might be related to the lack of robust evidence to support the efficacy of this type of training to prevent injuries or improve performance [73, 74]. As such, current research suggests that flexibility should be retired as a major component of physical fitness, to simplify and shorten training and testing routines and therefore, save the time and resources devoted to its instruction [75]. This could be even more relevant in Brazilian soccer, where congested fixtures and lack of appropriate resources together represent 63% of the biggest issues that SCCs have to face in their daily practices.

Most Brazilian SCCs (94%) implement dynamic, ballistic, and active forms of flexibility training, which appears to be driven by the consistent evidence in this regard revealing that: 1) static stretching does not enhance (and could even impair) strength and power performance, and 2) dynamic stretching may offer additional benefits for sport actions requiring power and agility (which are essential for soccer) [56, 59, 73]. Overall, about 70% of SCCs prescribe veryshort flexibility workouts (i.e., ≤ 15 minutes) and 20% of them do not include flexibility training sessions in their training programs. From these results, it may be suggested that Brazilian SCCs adopt practices in line with the current recommendations for flexibility training and development [73, 74].

### Physical Testing and Technology Use

All Brazilian SCCs regularly test their soccer players. Physical measurements are predominantly conducted all year round (53%) and during the pre-season (47%) and in-season (22%) phases. These data are slightly different from those obtained by Weldon et al. [14] who reported that 42% of soccer SCCs across different countries and leagues habitually apply physical tests during the competitive period, which represents an increase of 20% when compared to our results (22%, for the in-season phase). These discrepancies are likely due to differences in competitive schedules and other structural issues, which, as mentioned earlier, may greatly limit the time available for conducting complementary training and testing sessions during the Brazilian soccer season. A wide variety of physical tests are commonly used by the SCCs, such as body composition (96%), muscular strength (71%), muscular power (69%), anthropometry (63%), cardiovascular endurance (55%), linear and change of direction speed (47%), anaerobic capacity (43%), acceleration (24%), and flexibility (18%). Notably, the well-being of soccer players was consistently monitored by 100% of the Brazilian SCCs via online questionnaires or mobile applications (45%), verbal questionnaires (43%), and written questionnaires (31%). This is also a common and popular testing practice in other soccer leagues [14, 76], which can be easily understood by considering two basic aspects: 1) the close relationships already observed between subjective measures of perceived well-being and different performance and recovery outcomes in elite soccer players [76–78]; 2) the practical and inexpensive characteristics of well-being scales, which can be easily employed in different forms and contexts (e.g., immediately before and after training and matches) [77, 78]. Concerning the use of technology, GPS (88%), electronic jump mats (71%), heart rate monitors (67%), body composition analyzers (67%), and speed gates (41%) are among the most commonly used devices, which are very similar to the data reported for international SCCs [14]. The only marked difference between Brazilian SCCs and those from other countries is the less frequent use of force plates and bar-velocity trackers (e.g., linear position transducers) by the Brazilian SCCs (24% and 24% versus 50% and 35%, respectively, for force plates and bar-velocity trackers). The high costs of these devices (including high Brazilian import duties and unfavorable exchange rates) associated with the low number of local suppliers may explain, in part, the low utilization of these types of equipment in the Brazilian soccer scenario.

The authors recognize that this study is limited by the use of a standardized survey that includes a series of multiple-choice and open-ended responses, thus restricting the number and depth of questions asked. However, this is a common limitation of surveybased research which, on the other hand, allows for direct comparisons between different samples and populations.

## CONCLUSIONS

In general, the practices and strategies of Brazilian SCCs are highly influenced by the congested fixture schedules of Brazilian elite soccer. Moreover, these practices seem to be affected by the dimensions of the country and economic disparities between regions and clubs. Resistance training volume and frequency may be inappropriate (i.e., reduced) for significantly enhancing speed and power qualities; nevertheless, this is not an exclusive problem of Brazilian soccer. Some traditional and cultural aspects could potentially impact exercise selection and testing prescription leading to, for example, reduced use of Olympic lifts and 1RM tests among coaches. The vast majority of Brazilian soccer SCCs do not use periodization strategies to structure resistance training and prefer to organize and adjust training programs according to individual or collective responses to training and matches, without defining or predicting specific periods of peak performance. Speed training strategies are in line with current evidence for speed development, whereas plyometric practices should be revisited to improve training efficiency and quality. Flexibility development does not emerge as a relevant training objective in the Brazilian soccer context and, therefore, a substantial number of coaches do not prescribe flexibility training sessions or even use any type of flexibility exercise in their daily routines. All Brazilian SCCs regularly assess their athletes throughout the year and during the pre-season phase, but only 22% of them conduct physical measurements during the competitive period (which may reflect the congested fixture schedule of Brazilian soccer). Brazilian practitioners, the confederation, and federations can utilize the findings reported here to improve their strength and conditioning practices and create more specific and customized educational programs for Brazilian SCCs. The international soccer community and sport scientists can also use this information to design training programs more closely tailored to the background and needs of Brazilian professional soccer players, which may be essential to better (and faster) adapt them to different competitive scenarios and game demands, such as those found in the most important soccer leagues worldwide (e.g., *LaLiga*, Premier League, Italian Serie A League, etc.).

## Conflict of interest

The authors declare no conflict of interest.

## References

[1] Rocha CM, Fleury FA. Attendance of Brazilian soccer games: the role of constraints and team identification. Eur Sport Manag Q. 2017;17(4):485–505.

[2] Wachelke JFR. Brazilian fans’ social representations on soccer. (Representaciones sociales de los hinchas brasileños sobre fútbol). Rev Int Cienc Deporte. 2008;4(13):1–19.

[3] Brazilian Football Confederation. https://www.cbf.com.br/futebol-brasileiro/competicoes. Accessed February 23/2021.

[4] Brazilian Football Confederation: Raio X do Mercado: 2019. https://www.cbf.com.br/a-cbf/informes/index/raio-x-do-mercado-2019-numeros-gerais-de-registro. Accessed February 23/2021.

[5] Guedes SL. On criollos and capoeiras: notes on soccer and national identity in Argentina and in Brazil. Soccer Soc. 2014;15(1):147–161.

[6] Paulucio D. Monitoring of load in athletes of a league of the brazilian championship. Act Fis Deporte 2019;31:30–30.

[7] López AM. Brazil: receipts for international FIFA transfers 2013–2019. https://www.statista.com/statistics/1070065/fifa-transfer-revenue-brazil/. Accessed February 23/2021.

[8] Barnes C, Archer DT, Hogg B, Bush M, Bradley PS. The evolution of physical and technical performance parameters in the English Premier League. Int J Sports Med. 2014;35(13):1095–1100. 25009969 10.1055/s-0034-1375695

[9] Bush M, Barnes C, Archer DT, Hogg B, Bradley PS. Evolution of match performance parameters for various playing positions in the English Premier League. Hum Mov Sci. 2015;39:1–11. 25461429 10.1016/j.humov.2014.10.003

[10] Dellal A, Chamari K, Wong DP, Ahmaidi S, Keller D, Barros R, Bisciotti GN, Carling C. Comparison of physical and technical performance in European soccer match-play: FA Premier League and La Liga. Eur J Sport Sci 2011;11(1):51–59.

[11] George S, Ionel M, Cristian P. A comparative Study on the Evolution of the Parameters in Professional Soccer Matches. Procedia Soc Behav Sci. 2014;127:63–67.

[12] Loturco I, Pereira LA, Kobal R, Zanetti V, Gil S, Kitamura K, Abad CC, Nakamura FY. Half-squat or jump squat training under optimum power load conditions to counteract power and speed decrements in Brazilian elite soccer players during the preseason. J Sports Sci. 2015;33(12):1283–1292. 25772972 10.1080/02640414.2015.1022574

[13] Meckel Y, Harel U, Michaely Y, Eliakim A. Effects of a very short-term preseason training procedure on the fitness of soccer players. J Sports Med Phys Fitness. 2014;54(4):432–440. 25034547

[14] Weldon A, Duncan MJ, Turner A, Sampaio J, Noon M, Wong D, Lai VW. Contemporary practices of strength and conditioning coaches in professional soccer. Biol Sport. 2021;38(3):377–390. 34475621 10.5114/biolsport.2021.99328 PMC8329977

[15] Braun V, Clarke V. Using thematic analysis in psychology. Qualitative Res Psychol. 2006;3(2):77–101.

[16] Weldon A, Duncan MJ, Turner A, Christie CJ, Pang CMC. Contemporary practices of strength and conditioning coaches in professional cricket. Int J Sports Sci Coaching. 2020;In Press:1747954120977472.

[17] Loturco I, Kobal R, Kitamura K, Abad CCC, Faust B, Almeida L, Pereira LA. Mixed training methods: effects of combining resisted sprints or plyometrics with optimum power loads on sprint and agility performance in professional soccer players. Front Physiol. 2017;8:1034. 29311968 10.3389/fphys.2017.01034 PMC5732948

[18] Wong PL, Chaouachi A, Chamari K, Dellal A, Wisloff U. Effect of preseason concurrent muscular strength and high-intensity interval training in professional soccer players. J Strength Cond Res. 2010;24(3):653–660. 19816215 10.1519/JSC.0b013e3181aa36a2

[19] Loturco I, Pereira LA, Reis VP, Zanetti V, Bishop C, McGuigan MR. Traditional Free-Weight Vs. Variable Resistance Training Applied to Elite Young Soccer Players During a Short Preseason: Effects on Strength, Speed, and Power Performance. J Strength Cond Res. 2020;In Press 10.1519/JSC.0000000000003899 33298713

[20] Loturco I, Pereira LA, Reis VP, Bishop C, Zanetti V, Alcaraz PE, Freitas TT, McGuigan MR. Power training in elite young soccer players: Effects of using loads above or below the optimum power zone. J Sports Sci. 2020;38(11–12):1416–1422. 31389308 10.1080/02640414.2019.1651614

[21] Loturco I, Jeffreys I, Kobal R, Abad CCC, Ramirez-Campillo R, Zanetti V, Pereira LA, Nakamura FY. Acceleration and speed performance of Brazilian elite soccer players of different age-categories. J Hum Kinet. 2018;64: 205–218. 30429912 10.1515/hukin-2017-0195 PMC6231337

[22] Helgerud J, Rodas G, Kemi OJ, Hoff J. Strength and endurance in elite football players. Int J Sports Med. 2011;32(9):677–682. 21563031 10.1055/s-0031-1275742

[23] Carling C, McCall A, Le Gall F, Dupont G. What is the extent of exposure to periods of match congestion in professional soccer players? J Sports Sci. 2015;33(20):2116–2124. 26460635 10.1080/02640414.2015.1091492

[24] Dellal A, Lago-Peñas C, Rey E, Chamari K, Orhant E. The effects of a congested fixture period on physical performance, technical activity and injury rate during matches in a professional soccer team. Br J Sports Med. 2015;49(6):390–394. 23422422 10.1136/bjsports-2012-091290

[25] Julian R, Page RM, Harper LD. The Effect of Fixture Congestion on Performance During Professional Male Soccer Match-Play: A Systematic Critical Review with Meta-Analysis. Sports Med. 2021;51(2):255–273. 33068272 10.1007/s40279-020-01359-9 PMC7846542

[26] Loturco I, Pereira LA, Bishop C, Zanetti V, Freitas TT, Pareja-Blanco F. Strength-deficit in young soccer players: effects of a resistance training intervention. Biol Sport. 2022;39(3):615–619. 10.5114/biolsport.2022.106157 PMC9331327 35959330

[27] Coutts AJ. Fatigue in football: it’s not a brainless task! J Sports Sci. 2016;34(14):1296. 27049895 10.1080/02640414.2016.1170475

[28] Folgado H, Duarte R, Marques P, Sampaio J. The effects of congested fixtures period on tactical and physical performance in elite football. J Sports Sci. 2015;33(12):1238–1247. 25765524 10.1080/02640414.2015.1022576

[29] Jiménez-Reyes P, Garcia-Ramos A, Párraga-Montilla JA, Morcillo-Losa JA, Cuadrado-Peñafiel V, Castaño-Zambudio A, Samozino P, Morin JB. Seasonal Changes in the Sprint Acceleration Force-Velocity Profile of Elite Male Soccer Players. J Strength Cond Res. 2020;In Press. 10.1519/JSC.0000000000003513 32329976

[30] Lloyd RS, Oliver JL, Myer GD, De Ste Croix M, Read PJ. Seasonal variation in neuromuscular control in young male soccer players. Phys Ther Sport. 2020;42:33–39. 31869753 10.1016/j.ptsp.2019.12.006 PMC9892782

[31] Haugen TA. Soccer seasonal variations in sprint mechanical properties and vertical jump performance. Kinesiology. 2018;50(1):102–108.

[32] Saidi K, Zouhal H, Rhibi F, Tijani JM, Boullosa D, Chebbi A, Hackney AC, Granacher U, Bideau B, Ben Abderrahman A. Effects of a six-week period of congested match play on plasma volume variations, hematological parameters, training workload and physical fitness in elite soccer players. PLoS One. 2019;14(7):e0219692. 31344056 10.1371/journal.pone.0219692 PMC6657839

[33] Lundberg TR, Weckström K. Fixture congestion modulates post-match recovery kinetics in professional soccer players. Res Sports Med. 2017;25(4):408–420. 28795586 10.1080/15438627.2017.1365296

[34] Chad N. An approach to the periodisation of training during the in-season for team sports. Prof Strength Cond. 2010;18:5–10.

[35] Beato M, Maroto-Izquierdo S, Turner AN, Bishop C. Implementing Strength Training Strategies for Injury Prevention in Soccer: Scientific Rationale and Methodological Recommendations. Int J Sports Physiol Perform. 2021;In Press:1–6. 10.1123/ijspp.2020-0862 33503589

[36] Owen AL, Wong del P, Dellal A, Paul DJ, Orhant E, Collie S. Effect of an injury prevention program on muscle injuries in elite professional soccer. J Strength Cond Res. 2013;27(12):3275–3285. 23524368 10.1519/JSC.0b013e318290cb3a

[37] Daneshjoo A, Mokhtar AH, Rahnama N, Yusof A. The effects of comprehensive warm-up programs on proprioception, static and dynamic balance on male soccer players. PLoS One. 2012;7(12):e51568. 23251579 10.1371/journal.pone.0051568 PMC3520941

[38] Daneshjoo A, Mokhtar AH, Rahnama N, Yusof A. Effects of the 11 + and Harmoknee Warm-up Programs on Physical Performance Measures in Professional Soccer Players. J Sports Sci Med. 2013;12(3):489–496. 24149156 PMC3772593

[39] Marín-Pagán C, Blazevich AJ, Chung LH, Romero-Arenas S, Freitas TT, Alcaraz PE. Acute Physiological Responses to High-Intensity Resistance Circuit Training vs. Traditional Strength Training in Soccer Players. Biology (Basel). 2020;9(11) 10.3390/biology9110383 PMC7695212 33171830

[40] Dello Iacono A, Seitz LB. Hip thrust-based PAP effects on sprint performance of soccer players: heavy-loaded versus optimum-power development protocols. J Sports Sci. 2018;36(20):2375–2382. 29595081 10.1080/02640414.2018.1458400

[41] Loturco I, Nakamura FY, Kobal R, Gil S, Pivetti B, Pereira LA, Roschel H. Traditional periodization versus optimum training load applied to soccer players: effects on neuromuscular abilities. Int J Sports Med. 2016;37(13):1051–1059. 27706551 10.1055/s-0042-107249

[42] Loturco I, Suchomel T, Bishop C, Kobal R, Pereira LA, McGuigan M. One-Repetition-Maximum Measures or Maximum Bar-Power Output: Which Is More Related to Sport Performance? Int J Sports Physiol Perform. 2019;14(1):33–37. 10.1123/ijspp.2018-0255 29809068

[43] Rampinini E, Bosio A, Ferraresi I, Petruolo A, Morelli A, Sassi A. Match-related fatigue in soccer players. Med Sci Sports Exerc. 2011;43(11):2161–2170. 21502891 10.1249/MSS.0b013e31821e9c5c

[44] Bullock N, Martin DT, Ross A, Rosemond D, Marino FE. Effect of long haul travel on maximal sprint performance and diurnal variations in elite skeleton athletes. Br J Sports Med. 2007;41(9):569–573; 17473002 10.1136/bjsm.2006.033233 PMC2465388

[45] Fowler P, Duffield R, Vaile J. Effects of simulated domestic and international air travel on sleep, performance, and recovery for team sports. Scand J Med Sci Sports. 2015;25(3):441–451. 24750359 10.1111/sms.12227

[46] Calleja-Gonzalez J, Marques-Jimenez D, Jones M, Huyghe T, Navarro F, Delextrat A, Jukic I, Ostojic SM, Sampaio JE, Schelling X, Alcaraz PE, Sanchez-Bañuelos F, Leibar X, Mielgo-Ayuso J, Terrados N. What Are We Doing Wrong When Athletes Report Higher Levels of Fatigue From Traveling Than From Training or Competition? Front Psychol. 2020;11:194. 32153454 10.3389/fpsyg.2020.00194 PMC7046590

[47] Kataoka R, Vasenina E, Loenneke J, Buckner SL. Periodization: Variation in the Definition and Discrepancies in Study Design. Sports Med. 2021;In Press 10.1007/s40279-020-01414-5 33405190

[48] Stone MH, O’bryant HS, Schilling BK, Johnson RL, Pierce KC, Haff G, Koch AJ. Periodization: effects of manipulating volume and intensity. Part 1. Strength Cond J 1999;21(2):56.

[49] Jones RN, Greig M, Mawéné Y, Barrow J, Page RM. The influence of short-term fixture congestion on position specific match running performance and external loading patterns in English professional soccer. J Sports Sci. 2019;37(12):1338–1346. 30563419 10.1080/02640414.2018.1558563

[50] Ribeiro CC, Urrutia S. Scheduling the Brazilian soccer tournament with fairness and broadcast objectives. In, International Conference on the Practice and Theory of Automated Timetabling: Springer; 2006: 147–157

[51] Ribeiro CC, Urrutia S. Scheduling the Brazilian soccer tournament: Solution approach and practice. Interfaces. 2012;42(3):260–272.

[52] Chirosa IJ, Baena S, Soria MA, Bautista IJ, Chirosa LJ. Intra-repetition variable resistance training: part 1-an overview. Eur J Hum Mov. 2014;32:48–60.

[53] Wilson J, Kritz M. Practical guidelines and considerations for the use of elastic bands in strength and conditioning. Strength Cond J 2014;36(5):1–9.

[54] Ebben WP, Carroll RM, Simenz CJ. Strength and conditioning practices of National Hockey League strength and conditioning coaches. J Strength Cond Res. 2004;18(4):889–897. 15574099 10.1519/14133.1

[55] Simenz CJ, Dugan CA, Ebben WP. Strength and conditioning practices of National Basketball Association strength and conditioning coaches. J Strength Cond Res. 2005;19(3):495–504. 16095396 10.1519/15264.1

[56] Loturco I, Jeffreys I, Abad CCC, Kobal R, Zanetti V, Pereira LA, Nimphius S. Change-of-direction, speed and jump performance in soccer players: a comparison across different age-categories. J Sports Sci. 2020;38(11–12):1279–1285. 30724662 10.1080/02640414.2019.1574276

[57] Loturco I, Jeffreys I, Kobal R, Abad CCC, Ramirez-Campillo R, Zanetti V, Pereira LA, Nakamura FY. Acceleration and speed performance of Brazilian elite soccer players of different age-categories. J Hum Kinet. 2018;64:205–218. 30429912 10.1515/hukin-2017-0195 PMC6231337

[58] Loturco I, Kobal R, Maldonado T, Piazzi AF, Bottino A, Kitamura K, Abad CCC, Pereira LA, Nakamura FY. Jump squat is more related to sprinting and jumping abilities than Olympic push press. Int J Sports Med. 2017;38(8):604–612. 26667925 10.1055/s-0035-1565201

[59] Loturco I, Pereira LA, Kobal R, Maldonado T, Piazzi AF, Bottino A, Kitamura K, Cal Abad CC, Arruda M, Nakamura FY. Improving sprint performance in soccer: effectiveness of jump squat and Olympic push press exercises. PLoS One. 2016;11(4):e0153958. 27100085 10.1371/journal.pone.0153958 PMC4839661

[60] Haugen T, Tønnessen E, Hisdal J, Seiler S. The role and development of sprinting speed in soccer. Int J Sports Physiol Perform. 2014;9(3):432–441. 23982902 10.1123/ijspp.2013-0121

[61] Loturco I, Pereira LA, Filter A, Olivares-Jabalera J, Reis VP, Fernandes V, Freitas TT, Requena B. Curve sprinting in soccer: relationship with linear sprints and vertical jump performance. Biol Sport. 2020;37(3):277–283. 32879550 10.5114/biolsport.2020.96271 PMC7433323

[62] Lupo C, Ungureanu AN, Varalda M, Brustio PR. Running technique is more effective than soccer-specific training for improving the sprint and agility performances with ball possession of prepubescent soccer players. Biol Sport. 2019;36(3):249–255. 31624419 10.5114/biolsport.2019.87046 PMC6786321

[63] Loturco I, Pereira LA, Kobal R, Zanetti V, Kitamura K, Abad CC, Nakamura FY. Transference effect of vertical and horizontal plyometrics on sprint performance of high-level U-20 soccer players. J Sports Sci. 2015;33(20):2182–2191. 26390150 10.1080/02640414.2015.1081394

[64] Carlos-Vivas J, Perez-Gomez J, Eriksrud O, Freitas TT, Marin-Cascales E, Alcaraz PE. Vertical Versus Horizontal Resisted Sprint Training Applied to Young Soccer Players: Effects on Physical Performance. Int J Sports Physiol Perform. 2020;15(5):748–758. 32000140 10.1123/ijspp.2019-0355

[65] Pareja-Blanco F, Sáez de Villarreal E, Bachero-Mena B, Mora-Custodio R, Asián-Clemente JA, Loturco I, Rodríguez-Rosell D. Effects of Unloaded Sprint and Heavy Sled Training on Sprint Performance in Physically Active Women. Int J Sports Physiol Perform. 2020;In Press:1–7. 10.1123/ijspp.2019-0862 33004682

[66] Grazioli R, Loturco I, Lopez P, Setuain I, Goulart J, Veeck F, Inácio M, Izquierdo M, Pinto RS, Cadore EL. Effects of Moderate-to-Heavy Sled Training Using Different Magnitudes of Velocity Loss in Professional Soccer Players. J Strength Cond Res. 2020;In Press. 10.1519/JSC.0000000000003813 33009351

[67] Adams K, O’Shea JP, O’Shea KL, Climstein M. The effect of six weeks of squat, plyometric and squat-plyometric training on power production. J Appl Sport Sci Res 1992;6(1):36–41.

[68] Cormier P, Freitas TT, Rubio-Arias J, Alcaraz PE. Complex and Contrast Training: Does Strength and Power Training Sequence Affect Performance-Based Adaptations in Team Sports? A Systematic Review and Meta-analysis. J Strength Cond Res. 2020;34(5):1461–1479. 32084104 10.1519/JSC.0000000000003493

[69] Chu DA. Practical considerations for utilizing plyometrics. National Strength Cond Assoc J 1986;8(3):14–22.

[70] Comfort P, Matthews M. Strength and Conditioning. In: Comfort P, Abrahamson E eds, Sports Rehabilitation and Injury Prevention. Chichester: Wiley-Blackwell;2010: 223–244.

[71] Asadi A, Ramirez-Campillo R, Arazi H, Sáez de Villarreal E. The effects of maturation on jumping ability and sprint adaptations to plyometric training in youth soccer players. J Sports Sci. 2018;36(21):2405–2411. 29611771 10.1080/02640414.2018.1459151

[72] Paszkewicz J, Webb T, Waters B, Welch McCarty C, Van Lunen B. The effectiveness of injury-prevention programs in reducing the incidence of anterior cruciate ligament sprains in adolescent athletes. J Sport Rehabil. 2012;21(4):371–377. 22713252 10.1123/jsr.21.4.371

[73] Simic L, Sarabon N, Markovic G. Does pre-exercise static stretching inhibit maximal muscular performance? A meta-analytical review. Scand J Med Sci Sports. 2013;23(2):131–148. 22316148 10.1111/j.1600-0838.2012.01444.x

[74] Thacker SB, Gilchrist J, Stroup DF, Kimsey CD, Jr. The impact of stretching on sports injury risk: a systematic review of the literature. Med Sci Sports Exerc. 2004;36(3):371–378. 15076777 10.1249/01.mss.0000117134.83018.f7

[75] Nuzzo JL. The Case for Retiring Flexibility as a Major Component of Physical Fitness. Sports Med. 2020;50(5):853–870. 31845202 10.1007/s40279-019-01248-w

[76] Field A, Corr L, Thompson C, Gonzalez Lucena JC, Sarmento H, Naughton R, Brownlee T, Haines M, Page R, Harper L. Recovery following the extra-time period of soccer: Practitioner perspectives and applied practices. Biol Sport. 2022;39(1):171–179. 35173375 10.5114/biolsport.2022.104066 PMC8805349

[77] Kentta G, Hassmen P. Overtraining and recovery. A conceptual model. Sports Med 1998;. 26(1):1–16. 9739537 10.2165/00007256-199826010-00001

[78] Selmi O, Gonçalves B, Ouergui I, Sampaio J, Bouassida A. Influence of well-being variables and recovery state in physical enjoyment of professional soccer players during small-sided games. Res Sports Med. 2018;26(2):199–210. 10.1080/15438627.2018.1431540 29376416

